# Interdigital Neuroma in the Second Intermetatarsal Space Associated with Metatarsophalangeal Joint Instability

**DOI:** 10.1155/2016/9575917

**Published:** 2016-11-24

**Authors:** Takumi Matsumoto, Song Ho Chang, Naohiro Izawa, Yohei Ohshiro, Sakae Tanaka

**Affiliations:** Department of Orthopaedic Surgery, Faculty of Medicine, The University of Tokyo, 7-3-1 Hongo, Bunkyo-ku, Tokyo 113-8655, Japan

## Abstract

The entrapment theory is the most commonly accepted theory concerning the development of interdigital neuroma; it incriminates the deep transverse metatarsal ligament as the major causative factor of the condition. This report presents a patient with interdigital neuroma in the second intermetatarsal space, which was strongly suspected to be caused by the metatarsophalangeal joint instability due to plantar plate injury. Surgical intervention revealed that the neuroma was located more distally and dorsally than the deep transverse metatarsal ligament and was pinched between the adjacent metatarsal heads, suggesting the involvement of the metatarsophalangeal joint instability and chronic trauma as etiologies in this case.

## 1. Introduction

Interdigital neuroma in the foot, generally known as Morton's neuroma, is a painful condition that produces neuropathic pain in the distribution of the affected interdigital nerve [1]. Other conditions that cause forefoot pain can be differential diagnoses of Morton's neuroma and include stress fracture, metatarsalgia, degenerative and inflammatory arthritis, Freiberg disease, tarsal tunnel syndrome, tumors, and metatarsophalangeal (MTP) joint instability [1, 2]. Caution must also be exercised in case of the concomitant existence of these pathologies with interdigital neuroma. In particular, a high prevalence of coexisting second intermetatarsal space neuroma and second MTP joint instability has been reported [3]; however, there has been little discussion about the possible etiologic relationship between these two pathologies [3]. Because overlooking MTP joint instability can lead to residual pain after neurectomy for interdigital neuroma [4], it is important to understand the relationship between these two pathologies.

Here, we report a case of interdigital neuroma in the second intermetatarsal space accompanied with MTP joint instability of the second and third toes due to plantar plate rupture. The interdigital nerve was deflected dorsally and a spatulate-shaped neuroma was formed between the second and third metatarsal heads. The neuroma had a dent in the center, which was assumed to have been made by the impression between the metatarsal heads. The symptoms were completely resolved after neurectomy and plantar plate reconstruction of the second and third MTP joints. This case strongly supports the chronic trauma theory as one of the etiologies of interdigital neuroma and indicates a possible causal relationship between interdigital neuroma and MTP joint instability.

## 2. Case Report

The patient was a 62-year-old male with an 11-year history of dialysis due to chronic renal failure. He had complained of pain around the second and third metatarsal heads and numbness in the second and third lesser toes five to six years ago, which was accentuated by ambulation and shoes with a tight toe box. He had received conservative treatment including intermittent local analgesic injection a few years ago from other physicians and was referred to our hospital due to the gradual worsening of symptoms, which were unresponsive to conservative treatment.

**Figure 1:**
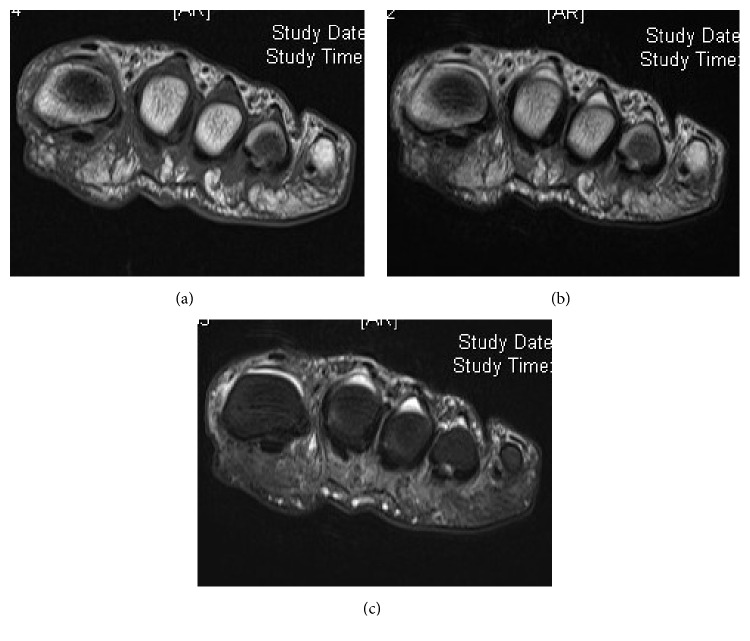
Magnetic resonance images of the distal metatarsals showing an upside-down bulbous-shaped mass with low-signal intensity in T1-weighted (a), T2-weighted (b), and short-tau inversion recovery image (c) extending dorsally between the second and third metatarsal heads.

When he presented at our hospital, he could not walk for more than 10 minutes at a time because of pain accentuated by ambulation. The static observation of the feet in the standing position showed hammertoe deformities of the second and third toes, which did not touch the ground. Clinical examination revealed tenderness to palpation in the second and third MTP joints and the second intermetatarsal space. The foot squeeze test examining Mulder's sign elicited a severe radiating pain but not the click [5]. The drawer test was positive in both the second and third MTP joints showing more than 50% subluxation and producing intolerable radiating pain in the second and third toes. Radiographs of the feet showed no apparent deviation of the toes but demonstrated a slight opening of the joint space at the second MTP joint. T1-weighted magnetic resonance imaging scans in the coronal plane showed an upside-down bulbous-shaped neuroma with low-signal intensity between the second and third metatarsal heads (Figure 1). Although taping in order to stabilize the toe in the neutral position was effective in reducing the pain accentuated by ambulation, it did not change the numbness in the toes. The taping was not continued for over a month by the patient because of discomfort; therefore, surgical treatment with excision of the interdigital neuroma and plantar plate repair was offered to the patient.

**Figure 2:**
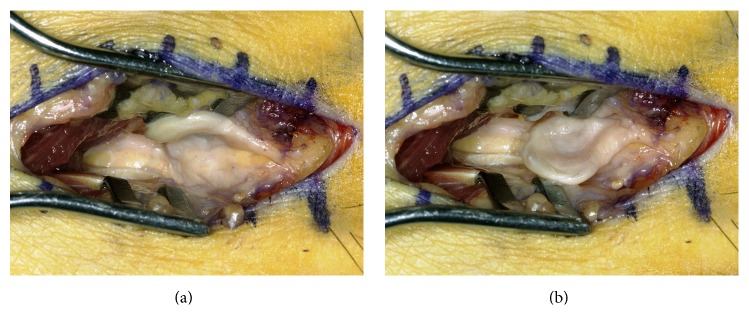
Intraoperative photograph. (a) Dorsally deviated course of the interdigital nerve and neuroma; (b) transversely reclined neuroma showing the impression in its center assumedly made by the adjacent metatarsal heads.

**Figure 3:**
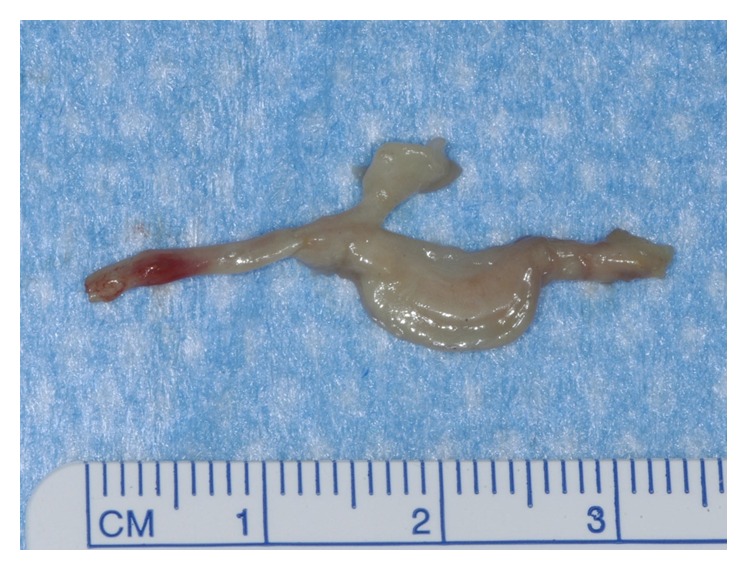
Excised interdigital nerve including a neuroma located distal to the bifurcation point.

The operation was performed under tourniquet control through a 3 cm dorsal incision over the second intermetatarsal space. Before dividing the deep transverse metatarsal ligament (DTML), the visibly enlarged interdigital nerve was observed which was deflected dorsally and was located between the second and third metatarsal heads (Figure 2(a)). The neuroma had a spatulate shape and had a dent in the center, which was plausibly due to the impression by the metatarsal heads (Figures 2(b) and 3). After the transection of the DTML, the second common digital nerve was dissected as proximally as practical before cutting it and any adjacent capsular nerve branches were served in order to allow the proximal nerve stump to retract into the intrinsic muscles of the foot. The nerve including the neuroma was resected with a length of 3 cm. Subsequently, the second and third MTP joints were exposed through the interval between the extensor digitorum longus and brevis tendons. The metatarsal head transected using Weil osteotomy was shifted proximally about 10 mm and temporarily fixed with a K-wire. An incomplete tear of the plantar plate was noted at the insertion of the plantar plate into the base of the proximal phalanx, which was a grade II tear in the second MTP joint with greater than 50% tear laterally and a grade I tear in the third MTP joint with less than 50% tear medially according to the grading by Nery et al. [6]. The plantar plates of the second and third MTP joints were repaired by sewing them back on the plantar base of the proximal phalanx following the method described by Watson et al. [7]. Shortly, a partially attached plantar plate was detached completely from the base of the proximal phalanx. A mattress suture was created in the plantar plate using the Mini Scorpion DX loaded with 0-Fiberwire (Arthrex, Naples, FL). Then, the sutures were retrieved into the two oblique holes which were made at the base of the proximal phalanx using a 0.062-inch K-wire. The K-wire in the Weil osteotomy was removed, and the metatarsal head was relocated in the position with 3 mm of shortening and fixed with an Asnis micro 2.0 mm cannulated titanium screw (Stryker Japan, Tokyo, Japan). Then, the sutures were tied over the dorsal aspect of the proximal phalanx with the toe plantar-flexed in 15 degrees at the MTP joint. Postoperatively, the patient was allowed to walk in a postoperative shoe with no weight on the forefoot for 3 weeks and with weight through the entire foot for another 3 weeks. Comfortable shoes and normal gait were permitted 6 weeks after the surgery. The histopathology result reported neural fibrosis, which was consistent with an interdigital neuroma.

The postoperative course was uneventful. At one year postoperatively, the patient could walk unrestricted in normal shoes. He had no pain but had a reduction of sensation in the supplying area of the resected nerve, which did not affect the patient's satisfaction. The Japanese Society for Surgery of the Foot (JSSF) ankle/hindfoot scale improved from 64 preoperatively to 85 one year postoperatively. The Self-Administered Foot Evaluation Questionnaire [8], which was developed and validated by JSSF as a patient-based outcome measure, improved or remained unchanged from the preoperative period to one year postoperatively in each subscale as follows: 71 to 81 in pain and pain-related, 75 to 89 in physical functioning and daily living, 83 to 88 in social functioning, 33 to 75 in shoe-related, and 100 to 100 in general health and well-being.

## 3. Discussion

Open neurectomy for interdigital neuroma is the most common procedure in patients with persistent symptoms unresponsive to the conservative treatment; however, some patients complain of continued forefoot pain even after the procedure. The rate of residual pain after neurectomy for interdigital neuroma has been reported to be from 4 to 35% [9–14]. While some of the continued pain after neurectomy might be attributed to inadequate resection or secondary conditions such as stump neuroma, some might be attributed to misdiagnosis or an overlooking of other concomitant pathologies. Haddad et al. reported that some cases with residual pain after neuroma resection in the second intermetatarsal space were successfully treated by flexor tendon transfer for correction of the crossover second toe deformity [4]. This study promotes awareness about concurrent MTP joint instability when treating interdigital neuroma. The study by Coughlin et al. involving 121 patients with symptomatic interdigital neuroma reported that 24 patients (20%) had a concurrent second MTP joint instability [3]. They also reported that 81% of these cases with two concurrent pathologies had neuromas in the second intermetatarsal space, a proportion markedly higher than in generally reported cases concerning the location of interdigital neuromas. The incidence of interdigital neuroma in the second intermetatarsal space has been reported to be from 5 to 32% [10, 11, 13, 15–18]. A reported higher prevalence of the second intermetatarsal space neuroma in cases with the second MTP joint instability compared to the prevalence of the second intermetatarsal space neuroma in the general population strongly suggests an association between these two pathologies.

Many histological studies have demonstrated that interdigital neuroma is not a true neuroma but a perineural fibrosis [15, 19–21]. Several theories have been advocated as etiologies of interdigital neuroma including the chronic repetitive trauma theory, entrapment theory, ischemic theory, and intermetatarsal bursitis theory [22]. Among these, the entrapment theory is the most common where the neuroma is considered to be formed by the entrapment of the interdigital nerve at the point where the nerve passes underneath the DTML [23, 24]. This theory is supported by the success of Gauthier's surgical technique consisting of the release of the anterior edge of the DTML without resection of the neuroma [23]. In 206 patients treated with this technique, 83% reportedly had rapid and stable improvement [23]. On the other hand, the chronic repetitive trauma theory suggests that repeated trauma to the interdigital nerve by mechanical effects during walking causes neuroma [15, 25]. The frequent involvement of the third common digital nerve has been explained anatomically since the third common digital nerve is generally formed by branches of the medial and lateral plantar nerves, which possibly make the nerve vulnerable to tethering [26]. Electron microscopic evaluation of the resected specimens of interdigital neuroma reported edema of the endoneurium, fibrosis beneath the perineurium, axonal degeneration, and necrosis, which suggested nerve damage secondary to mechanical impingement by adjacent structures [27]. While the DTML is necessarily involved in the pathology of the interdigital neuroma as demonstrated by the success of Gauthier's surgical technique, some authors have advocated the involvement of the metatarsal heads [5, 25, 28]. An anatomical study examining the relationship between the location of interdigital neuromas and the DTML demonstrated that the interdigital neuromas were located more distally than the DTML in both the mid-stance and the heel-off stage during walking, which raised an objection against an entrapment by the DTML and supported chronic repetitive trauma by the metatarsal heads as an etiology [28]. The eliciting procedure known as Mulder's click, which has high sensitivity and specificity in diagnosing interdigital neuroma, is considered to replicate the compression of neuromas by the metatarsal heads [5, 29, 30]. The neuroma in our case was located between the metatarsal heads distally apart from the DTML and showed the peculiar configuration suggesting mechanical compression by the adjacent metatarsal heads.

**Figure 4:**
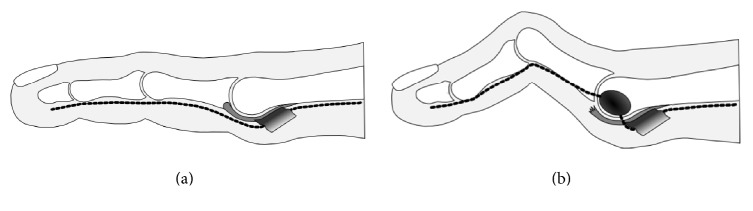
Diagram showing the etiology of interdigital neuroma associated with metatarsophalangeal joint instability. (a) The interdigital nerve runs consistently along the bottom side of the toe passing under the deep transverse metatarsal ligament at the distal metatarsal. (b) Metatarsophalangeal joint instability can curve the interdigital nerve with the distal end of deep transverse metatarsal ligament as a flexion point predisposing the nerve to impingement by the adjacent metatarsal heads.

Like the collateral ligaments, the plantar plate is a major static-stabilizing structure of the lesser MTP joint [6, 31] and its rupture has been well-documented as a primary pathology of MTP joint instability [31–33]. The plantar plate originates from the proximal neck of the metatarsal metaphysis with a thin synovial attachment and inserts distally into the plantar base of the proximal phalanx with a firm fibrocartilaginous attachment. Several structures have an attachment with the plantar plate including the plantar fascia, tendon sheath of the flexor tendons, DTML, collateral ligaments, and the interossei tendons [33, 34]. The insertion area of the DTML into the plantar plate is located at its proximal two-thirds and plantar one-third [34]. Although a plantar plate tear can be caused by trauma, most of the tears occur as degenerative changes around its distal attachment to the proximal phalanx [6]. A cadaveric study transecting the plantar plate at the distal attachment demonstrated an increase of displacement of the proximal phalanx by over 70% [31]. Clinically, the severest stage of plantar plate tear is even associated with dorsal dislocation of the proximal phalanx [6, 35]. Considering the course of the interdigital nerve under the DTML, MTP joint instability due to the plantar plate insufficiency is expected to cause pulling or dorsal deviation of the interdigital nerve with tethering at the distal end of the DTML (Figure 4). As plantar plate insufficiency occurs most commonly at the second MTP joint, the interdigital nerve most likely affected will be the one running through the second intermetatarsal space [6, 36].

When MTP joint instability and interdigital neuroma coexist, it is not easy to confirm which pathology produces the patient's chief complaint. Patients with interdigital neuroma generally complain of a burning pain localized to the plantar aspect of the forefoot, which may radiate into the toes. Conversely, patients with plantar plate insufficiency demonstrate a hammer toe or crossover toe if the deformity progresses; however, they only complain of a pain localized to the plantar aspect of the forefoot mimicking interdigital neuroma in the early stage with no toe deformity. Sequential xylocaine injections may help to differentiate the two pathologies; however, the possible spreading of injected fluid around adjacent structures makes the interpretation of results ambiguous [3, 37]. Recently, the direct repair of plantar plate rupture through a dorsal approach has been established with good clinical outcome [6, 7, 38]. The present case was successfully treated with neurectomy and direct repair of the plantar plate with no residual pain or toe deformity. We recommend that MTP joint instability accompanied with the interdigital neuroma should be treated with neurectomy in order to exclude the risk of residual pain or toe deformity progression.

## 4. Conclusion

We reported a case of interdigital neuroma in the second intermetatarsal space which could be attributed to the MTP joint instability. Considering the possible causal relationship between these two pathologies, MTP instability should be examined for carefully in patients diagnosed with interdigital neuromas, especially in neuromas occurring in the second intermetatarsal space.
